# Prevalence of, and factors associated with oral sexual behaviours in men and women in Ibadan, Nigeria

**DOI:** 10.1371/journal.pone.0328454

**Published:** 2025-07-16

**Authors:** Imran Oludare Morhason-Bello, Adekunle Daniel, Akinyele Adisa, Kathy Baisley, Isaac Adewole, Rasheed Bakare, Robert Murphy, Lifang Hou, Silvia de Sanjosé, Suzanna C. Francis, Deborah Watson-Jones

**Affiliations:** 1 Obstetrics and Gynaecology Department, Faculty of Clinical Sciences, College of Medicine, University of Ibadan, Ibadan, Nigeria; 2 Institute of Advance Medical Research and Training, College of Medicine, University of Ibadan, Ibadan, Nigeria; 3 Department of Otolaryngology, Faculty of Clinical Sciences, College of Medicine, University of Ibadan, Ibadan, Nigeria; 4 Department of Oral Pathology, Faculty of Dentistry, College of Medicine, University of Ibadan, Ibadan, Nigeria; 5 Department of Infectious Disease Epidemiology, Faculty of Epidemiology and Population Health, London School of Hygiene and Tropical Medicine, London, United Kingdom; 6 Department of Microbiology, Faculty of Basic Medical Sciences, College of Medicine, University of Ibadan, Ibadan, Nigeria; 7 Institute for Global Health, Northwestern University, Chicago, Illinois, United States of America; 8 Preventive Medicine Department, Cancer Epidemiology and Prevention, Northwestern University, Chicago, Illinois, United States of America; 9 Division of Cancer Epidemiology and Genetics (DCEG), National Cancer Institute (NCI), National Institutes of Health (NIH), USA and Associate Researcher, ISGlobal, Barcelona, Spain; 10 Mwanza Intervention Trials Unit, National Institute for Medical Research, Mwanza, Tanzania; 11 Clinical Research Department, Faculty of Infectious and Tropical Diseases, London School of Hygiene and Tropical Medicine, London, United Kingdom; Tarbiat Modares University, IRAN, ISLAMIC REPUBLIC OF

## Abstract

**Background:**

Unprotected oral sex is associated with sexually transmitted infections (STIs) including HPV and associated head and neck cancers. However, many studies lack clear definitions of oral sex and there are few data from West Africa. This study assessed the pattern and prevalence of, and factors associated with oral sex among Nigerian men and women from the general population.

**Methods:**

The Sexual Behaviours and HPV Infections in Nigerians in Ibadan (SHINI) study was a cross-sectional study conducted among men and women aged 18–45 years. Information on oral sexual behaviours was collected during a face-to-face interview by a sex-matched interviewer. Descriptive and multivariable analyses were used to analyse factors associated with oral sex in men and women.

**Results:**

In total, 160/626 (26%) had ever any type of oral sex. Specifically, 78/626 (12%) had ever given and 139/626 (22%) had ever received oral sex in a heterosexual relationship. Overall, 35% (112/310) men had ever engaged in oral sex compared to women 15% (48/316 –p < 0.001). More men frequently reported ever receiving oral sex than women (33% vs. 12%; p < 0.001). There was no difference in the proportion of men and women that reported ever giving oral sex. Most (94%) participants never used any barrier protection during oral sex. Most men and women participants engaged in oral sex because of romantic relationship or being married. In the adjusted models, there was a higher odds of ever receiving oral sex (Adjusted odds ratio = AOR 4.01 95% CI 2.52–6.38) or engaging in any form of oral sex (AOR = 3.12, 95%CI 2.04–4.76) in men than women.

**Conclusion:**

Though, Nigerian men and women engage in oral sex in heterosexual relationship, but it is more commonly reported by men than women and most of them practiced it without barrier protection. It is recommended that sexually active men and women are counselled on risks associated with unprotected oral sex including STIs, HIV and HPV and associated cancers.

## Background

Oral sex is defined as a sexual behaviour when a person uses his/her mouth or tongue touch the genitals or anus of another person. Cunnilingus involves licking or sucking the clitoris, vulva or vagina, while fellatio refers to licking or touching of penis and or scrotum and anilingus is sexual stimulation of the anus using the tongue [1]. Unprotected oral sex is associated with acquisition and transmission of sexually transmitted infections (STI) such as Neisseria gonorrhea, Chlamydia trachomatis, Hepatitis B, Herpes simplex, and human papillomavirus (HPV) amongst others. [2–7]. Oral sexual practices without barrier contraceptive methods increase the risk of oral HPV infection acquisition and transmission and has a direct association with the persistence of high-risk HPV, oral potentially malignant disorders (OPMD), and incidence of HPV-associated cancers of oral and oropharyngeal sites [4,8–11]. Generally, health risks associated with oral sexual behaviours depend on the role played by individuals during the sexual act [8]. For example, a woman or man giving oral sex is believed to be at a higher risk of acquiring STI and HIV than the person receiving it [8]. Risky oral sexual practices are more commonly reported in adolescents and younger adults relative to older adults, in men than in women and in people involved in same sex relationships than those involved in heterosexual relationships [12,13].

In over two decades, studies in high income countries such as the United States of America (USA), United Kingdom (UK), Europe and Australia have shown an increase in the prevalence of oral sex in addition to penetrative vaginal sex particularly, among young people, female sex workers and men who have sex with men [14–16]. In Europe, UK and the USA, the prevalence of ever performing oral sex (given or received) ranges from 67–94% in adolescents and adults with higher proportion in men compared to women [15,17–19]. The National Health and Nutrition Survey (NHANES) between 2009–10 found that of 2,116 men and 2,140 women, 85% of men and 83% of women had ever performed oral sex [15]. Additionally, the prevalence of ever performing oral sex in the analysis was significantly higher (90.3%) among the younger age group (30–44 years) compared with older age group and decreased with age [15]. A study in UK compared trends of fellatio and cunnilingus using the National Survey of Sexual Attitudes and Lifestyles (NATSAL 1, 2, and 3) among young people aged 16–24 years from 1990–2012 [20]. The findings showed that the proportion of male and female participants that reported fellatio increased from the first (66% vs 64%) to second (72% vs 73%) and third (75% vs 73%) surveys [20]. A similar report was noted on cunnilingus from 67% in males and 66% in females in NATSAL 1–69% and 74% in NATSAL 2, and 71% and 72% in NATSAL 3, in males and females respectively [20]. In another study, a similar trend of increasing report of oral sexual behaviours was reported among adults across the three NATSAL surveys [17].

In 2019, a systematic review involving 103 articles from Sub-Saharan Africa (SSA) showed that oral sexual behaviours, largely without barrier contraceptive methods, were commonly reported among young people and female sex workers involved in heterosexual relationships [21]. In the same review, the prevalence of oral sex in younger population particularly among the university students was up to 46% and 80% among female sex workers relative to less than 40% among adults in general population. Less than 17% ever used condom or any barrier methods during oral sex. Furthermore, only four out of 103 studies reviewed clearly defined and correctly classified oral sex by the actions of participants (given or received) [21]. This makes it difficult to appreciate potential health risks associated with the oral sex reported in those studies. Despite the increasing reports of oral sex in adolescents and adults involved in heterosexual relationships in Nigeria and many countries in West Africa, none of these studies was conducted at the population level and adequately powered for a generalizable conclusion [21,22]. This study aimed to determine the pattern and prevalence of, and factors associated with oral sex among men and women in the general population using a validated tool.

## Methods

### Study design, population, and site

This study analyses data collected from the Sexual Behaviours and HPV Infections in Nigerians in Ibadan (SHINI) study [23]. Briefly, the SHINI study was a mixed methods study that explored the association between different sexual behaviours and the human papillomavirus infections in men and women aged 18–45 years from the general population in Ibadan metropolis and in brothel-based female sex workers (FSW) of the same age in Ibadan. The methods for this study have been previously described [23]. In brief, SHINI was a cross-sectional household survey conducted among 315 males and 310 females aged 18–45 years living in Moniya and Sasa in Akinyele and Mokola in Ibadan North LGAs and 316 FSW recruited from brothels in LGAs within Ibadan metropolis. The study excluded young adolescents (<18 years), pregnant or nursing mothers, women not resident at the study sites and those that declined participation including refusal for biological sample collection.

Men and women in the general population were recruited by using a two-stage sampling technique. In the first stage sampling, 4 EAs in Ibadan North while 5 EAs in Akinyele LGAs were selected by probability proportion to size from the list of National Population Commission Census [24]. Houses located in the 9 selected EAs were listed and a census of participants aged 18–45 years living in those houses was done to form the sampling frame. A systematic random sampling technique was used in the second stage to select eligible males and females in the general population. The FSWs selected by simple random sampling in brothels that had 11 persons while total sampling was done for brothels that had 10 or less persons. The detailed descriptions of sampling techniques had been published previously [25–27].

Prior to this study, research assistants and nurses had two intensive trainings on research methods including good clinical practices, research ethics and study protocol. After, a pilot study was conducted to test the research tools and for any possible revisions.

Male and female participants consented to face-to-face interviews by trained sex matched research assistants, a physical examination and collection of biological samples from penile, cervical, vulva, anal and oral cavity by sex matched research nurses for HPV genotyping [23,25–27]. Participants were seen at primary healthcare facilities in their communities. The original sample size of 300 was calculated based on the assumption of an alpha of 0.05 and a design effect of 2 for the clustered sampling design, to be able to estimate the prevalence of HPV and determine associated risk factors in each of the four anatomic sites. The determination of oral sex was a secondary outcome in the SHINI study.

### Data source

Data for the analyses of oral sex were taken from the general population of men and women including FSWs in the brothels. In this study, we used validated definitions of oral sex from the in NATSAL survey [28]. Oral sex was defined as using the mouth or tongue to touch any parts of the genital area of a sexual partner. A person was reported to have given oral sex when he/she used his/her mouth or tongue to touch any part of the genital area of the sexual partner. Ever receiving oral sex was defined as a history of allowing a sexual partner to touch the partner’s genital area with his/her mouth or tongue. Briefly, we extracted information on sociodemographic characteristics, sexual relationships including oral sex, partnerships, lifestyle practices (alcohol consumption and tobacco and illicit drug use), awareness of HPV and any report of ever having had an HIV test.

### Data management

We conducted a double data entry with REDCap software (Vanderbilt University, Nashville Tennessee, USA). The data were exported and imported into STATA 16.0 (Stata 2019. Statistical Software: Release 16. College Station, TX: StataCorp LLC) software for analysis. Exploratory analysis involved description of frequencies and proportions for categorical variables and mean and standard deviations for continuous variables to check for missingness and implausible responses.

**Fig 1:**
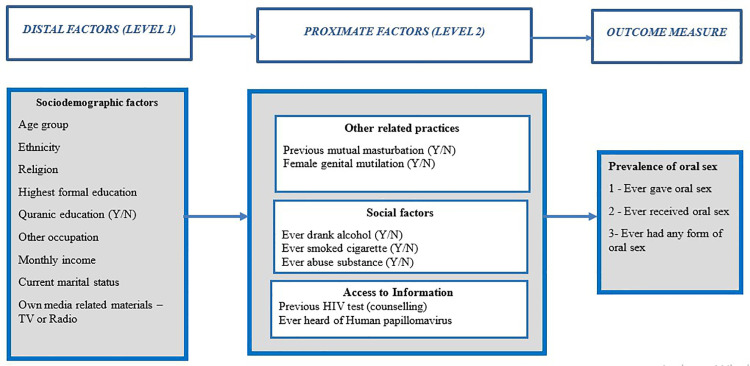
Conceptual Framework for the risk factor analysis of oral sex among sexually active men and women in Ibadan, Nigeria.

The primary outcome was any report of ever had oral sex (yes/no) among men and women in this study. We calculated the prevalences of ever having oral sex and ever giving or receiving oral sex by men and women. A conceptual framework for the assessment of associated factors was developed (Fig 1).

Due to the small numbers of observations, we performed analysis of factors associated with ever having oral sex by combining data on ever giving or receiving oral sex. Univariate and multivariable logistic regression models were performed to obtain unadjusted and adjusted estimates using a hierarchical modelling approach for ever having oral sex. The sex of participants (level 1 factor) was included in the adjusted estimates a priori. Each variable of sociodemographic characteristics (level 2 factors – age, ethnicity, religion, highest formal education, quranic education (y/n), other occupation, monthly income, current marital status, own media related materials – TV or Radio) was added one by one to the model that included sex and p-values obtained by likelihood ratio tests. Variables with a p value ≤ 0.10 were included in the adjusted model. Next, any proximate factors (Level 3 – history of masturbation, female genital mutilation, history of alcohol intake and cigarette, HIV test and knowledge of HPV) that met a p value cut off ≤ 0.1 was included with the level 1 and 2 ‘core variables’ in the level 3 adjusted model.

### Ethics approval and consent to participate

Each participant signed written or witnessed informed consent after reading the information leaflet including the objectives and procedures of the study with clarifications by the research assistants. Participants were assured of confidentiality and anonymity on their response including data provided. Specifically, each participant had a face-to-face interview in a private room in the clinic with a sex-matched research assistant, and case report forms had no identifier and were kept in a locked cabinet in the office of principal investigator with restricted access.

Ethical approvals were obtained from the ethical committees of the London School of Hygiene and Tropical Medicine, London (LSHTM 9736); the University of Ibadan/the University College Hospital, Ibadan (UI/EC/16/005); and the Oyo State Government (AD13/479/712) in Nigeria. The team received concurrence of the leadership of Ibadan North and Akinyele LGAs and owners of the brothels where the study was conducted. The recruitment and data collection of quantitative data of SHINI study was conducted between 23^rd^ of March and 30^th^ July 2018.

### Inclusivity in global research

Additional information regarding the ethical, cultural, and scientific considerations specific to inclusivity in global research is included in the **Supporting Information (**S1 Checklist**).**

## Results

### Participant characteristics

A total of 626 participants, 310 (49.5%) women and 316 (50.5%) men, were included in the study. The median age of men and women was 26 years with interquartile ranges of 21–35 years and 23–35 years, respectively. There were slightly higher proportions of men (52% vs 48%) and women (51% vs 49%) in Ibadan North than in Akinyele LGAs. There were more men (72%) with secondary education than women (58%) (p < 0.001). Women had higher proportions of semi-skilled workers relative to men (72% vs 64%), but there were more skilled people among men than in women (14% vs 6%). A little above half (52%) of women and a third of men (33%) either earned no income or less than 10,000 Naira (<28 USD) per month (p < 0.001). More men were single and living alone compared to women (63% vs 27%). There were more men that personally owned mobile phone (95% vs 85%), television (75% vs 52%), and radio (64% vs 40%) relative to women (p < 0.001) (Table 1).

**Table 1:** Socio-demographic characteristics of sexually active men and women in two communities in Ibadan, Nigeria.

Variable	Total N = 626	Female N = 310	Male N = 316	p-value
	N (% column)	N (%column)	n (% column)	
**Age, years**				
Median (IQR)	26 (22-35)	26 (23-35)	26 (21-35)	0.319
**Age group, years**				
18-24	259 (41%)	121 (39%)	138 (44%)	
25-34	198 (32%)	101 (33%)	97 (31%)	0.489
35-45	169 (27%)	88 (28%)	81 (26%)	
**Study Site** ^**1**^				
Ibadan North	320 (51.1%)	157 (50.7%)	163 (51.6%)	0.408
Akinyele	306 (48.9%)	153 (49.4%)	153 (48.4%)	
**Ethnicity**				
Yoruba	485 (77%)	240 (77%)	245 (78%)	
Hausa/Fulani	51 (8%)	37 (12%)	14 (4%)	
Igbo	40 (6%)	19 (6%)	21 (7%)	<0.001
Other ethnic groups	50 (8%)	14 (5%)	36 (11%)	
**Religion**				
Christian	273 (44%)	140 (45%)	133 (42%)	
Islam	351 (56%)	168 (54%)	183 (58%)	0.272
Traditional	2 (0%)	2 (1%)	0 (0%)	
**Highest education level**				
Primary level or None	72 (12%)	56 (18%)	16 (5%)	
Any secondary level	402 (65%)	176 (58%)	226 (72%)	<0.001
Any tertiary level	145 (23%)	72 (24%)	73 (23%)	
**Occupation**				
No current paid job (e.g., student, housewife)	111 (18%)	51 (16%)	60 (19%)	
Unskilled worker (e.g., office assistant, food vendor)	23 (4%)	15 (5%)	8 (3%)	0.004
Semi-skilled worker (e.g., driver, tailor)	427 (68%)	224 (72%)	203 (64%)	
Skilled worker (e.g., teacher, technician, doctor)	65 (10%)	20 (6%)	45 (14%)	
**Income per month** ^**2**^				
No income	71 (11%)	35 (11%)	36 (11%)	
Less than 10,000N (1–28USD)	197 (31%)	126 (41%)	71 (22%)	<0.001
10,000–20,000N (>29–56USD)	153 (24%)	85 (27%)	68 (22%)	
More than 20,000N (>56USD)	205 (33%)	64 (21%)	141 (45%)	
**Current marital status**				
Single and Living alone	283 (45%)	84 (27%)	199 (63%)	
Married and living as married	318 (51%)	210 (68%)	108 (34%)	<0.001
Divorced/widowed/separated and living alone	25 (4%)	16 (5%)	9 (3%)	
**Items personally owned by participant**				
Mobile phone	565 (90%)	265 (85%)	300 (95%)	<0.001
Television	398 (64%)	161 (52%)	237 (75%)	<0.001
Radio	327 (52%)	124 (40%)	203 (64%)	< 0.001
Generator	188 (30%)	77 (25%)	111 (25%)	0.005
House	33 (5%)	14 (5%)	19 (6%)	0.402

*1-LGA – Local Government Area; 2–357 Naira (Nigeria National Currency) = 1 USD as at the time the study was conducted*

**Fig 2:**
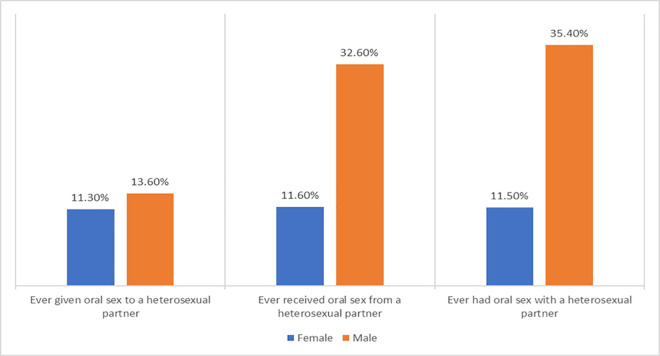
Pattern of oral sexual behaviour of sexually active men and women in two communities in Ibadan, Nigeria.

There were more men (112/316–35%) compared to women (48/310–15%) that had ever engaged in any form of oral sex with a heterosexual partner (p < 0.001) (Table 2; Fig 2).

**Table 2:** Sexual relationships and behaviours in two communities in Ibadan, Nigeria.

Variable	Total N = 626	Female N = 310	Male N = 316	p-value
	n (% column)	n (% column)	n (% column)	
**Currently in a sexual relationship**				
No	41 (7%)	12 (4%)	29 (9%)	0.007
Yes	585 (93%)	298 (96%)	287 (91%)	
**Age of current main sexual partner, years** ^1^				
Median (IQR)	30 (22-38)	35 (30-43)	23 (19-29)	<0.001
**Ever gave oral sex to a heterosexual partner**				
No	548 (88%)	275 (89%)	273 (86%)	0.380
Yes	78 (12%)	35 (11%)	43 (14%)	
**Ever received oral sex from a heterosexual partner**				
No	487 (78%)	274 (88%)	213 (67%)	<0.001
Yes	139 (22%)	36 (12%)	103 (33%)	
**Ever had oral sex with a heterosexual partner (given or received combined)**				
No	466 (74%)	262 (85%)	204 (65%)	<0.001
Yes	160 (26%)	48 (15%)	112 (35%)	
**Median Age (SD) when first oral sex was given to a heterosexual partner, years** ^2^				
Median (IQR)	25 (20-32)	25 (22-32)	26 (20-32)	0.108
**Age when first oral sex was received from a heterosexual partner** ^**3**^ **, years**				
Median (IQR)	24 (19-28)	24 (21.5-29)	23 (18-28)	0.028
**Age of partner when first oral was received** ^**3**^ **, years**				
Median (IQR)	23 (18-28)	30 (26.5-36.5)	20 (17-25)	<0.001
**Age of partner when first oral sex was given** ^**4**^ **, years**				
Median (IQR)	25 (20-32)	31 (27-37)	22 (18-26)	<0.001
**Number of lifetime partners given oral sex** ^4^				
Single oral sex partner	55 (71%)	29 (83%)	26 (60%)	0.031
Multiple oral sex partners (≥ 2)	23 (29%)	6 (17%)	17 (40%)	
**Any use of barrier method by partner when oral sex was last given** ^, 5^				
No	625 (100%)	310 (100%)	315 (99%)	1.000
Yes	1 (0%)	0 (0%)	1 (0%)	
**Number of lifetime partners you have received oral sex from** ^6^				
Single oral partner	96 (69%)	30 (83%)	66 (64%)	0.031
Multiple oral sex partners (≥ 2)	43 (31%)	6 (17%)	37 (36%)	
**Any use of barrier methods during the last time oral sex was given** ^5,7^				
No	73 (94%)	33 (94%)	40 (93%)	1.000
Yes	5 (6.4%)	2 (6%)	3 (7%)	
**Any barrier methods used during last time oral sex was received** ^5,6^				
No	136 (98%)	34 (94%)	102 (99%)	0.164
Yes	3 (2%)	2 (6%)	1 (1%)	
**Ever had anal sex** ^5^				
No Yes	609 (97%) 17 (3%)	309 (100%) 1 (0%)	300 (95%) 16 (5%)	<0.001
**Age at first vaginal sex, years** ^8^				
≤ 15	91 (15%)	34 (11%)	57 (18%)	<0.001
16-17	112 (18%)	42 (14%)	70 (22%)	
18-24	357 (57%)	202 (65%)	155 (49%)	
≥ 25	65 (10%)	32 (10%)	33 (10%)	
**Age of first vaginal sex partner, years** ^8^				
Median (IQR)	18 (17-21)	19 (18-21)	18 (16-20)	0.040
**Number of lifetime partners for vaginal sex** ^8^				
Single vaginal partner	220 (35%)	163 (53%)	57 (18%)	<0.001
Multiple vaginal sex partners (≥ 2)	405 (65%)	147 (47%)	258 (82%)	
**Condom use during last vaginal sex** ^8^				
No	468 (75%)	254 (82%)	214 (68%)	<0.001
Yes	157 (25%)	56 (18%)	101 (32%)	
**Ever had transactional sex**				
No	593 (95%)	291 (94%)	301 (95%)	0.446
Yes	34 (5%)	19 (6%)	15 (5%)	
**Condom use for last transactional sex** ^9^				
No	18 (53%)	11 (58%)	7 (47%)	0.515
Yes	16 (47%)	8 (42%)	8 (53%)	
**Ever practiced mutual masturbation** ^8,10^				
No	145 (23%)	76 (25%)	69 (22%)	0.439
Yes	480 (77%)	234 (75%)	246 (78%)	
**Ever practiced self-masturbation**				
No	230 (37%)	135 (44%)	95 (30%)	
Yes	396 (63%)	175 (56%)	221 (70%)	< 0.001
**Has undergone male circumcision/female genital mutilation** ^**11**^				
No	128 (20%)	120 (39%)	8 (3%)	<0.001
Yes	498 (80%)	190 (61%)	308 (97%)	
**Ever consumed alcohol.**				
No	334 (53%)	224 (72%)	110 (35%)	
Yes	292 (47%)	86 (28%)	206 (65%)	< 0.001
**Ever smoked cigarettes.**				
No	531 (85%)	304 (98%)	227 (72%)	<0.001
Yes	95 (15%)	6 (2%)	89 (28%)	
**Ever taken any illicit drugs** ^12^				
No	489 (78%)	288 (93%)	201 (64%)	<0.001
Yes	137 (22%)	22 (7%)	115 (36%)	

*1-****1****- 41 missing; 2–548- missing;* ***3–****487 missing; 4–548- missing;* ***5-*** *Fischer exact test; 6****–****487 missing; 7–548- missing; 8–1 missing; 9–592 missing; 10-****-*** *Mutual masturbation question was ‘have you or your partner ever touched each other’s genital area by hand? (Yes or No); 11- Female genital mutilation was based on the clinical examination of the female external genitalia for evidence of genital circumcision by the research nurse at the clinic (Yes or No) and* ***-****Genital circumcision was defined as female genital mutilation or removal of prepuce skin from the penile shaft;* ***12-****Illicit drugs are banned substances or drugs taken by participants for non-medical reasons in Nigeria*

Furthermore, there were more reports of fellatio than cunnilingus (33% vs 12%, p < 0.001). Thirty-four out of 316 (11%) men and 23/310 (7%) women had engaged in both fellatio and cunnilingus, but 204/316 (65%) men and 262/310 (85%) had never had fellatio or cunnilingus (*data not shown*). The median age at first oral sex was received was lower in men than women (p = 0.028), but there was no significant difference age at first oral sex was given in the two populations (p = 0.108) (Table 2). Men reported having more multiple lifetime oral sex partners relative to women (40% vs 17%, p = 0.031).

In the past three months, over a third of women had given (13/36; 37%) and had received (13/35; 36%) oral sex. Over the same period, more men had received oral sex (27/103; 26%) than gave oral sex (7/43; 16%) with their partners. Only very few participants had ever used barrier methods while giving or receiving oral sex in men and women. There were more men that ever-practiced anal sex relative to women (5% vs 0%, p < 0.001) (Table 2). Regarding penile-vaginal sex practices, the current age of the main sexual partner was younger in men than women (p < 0.001). Men reported a relatively younger age at first penile-vaginal sex than women (p < 0.001) and more men than women reported having multiple lifetime sexual partners (p < 0.001). More men than women had history of self-masturbation (p < 0.001), circumcision (p < 0.001), alcohol intake (p < 0.001), cigarette (p < 0.001) and illicit drug use (p < 0.001).

### Reasons for engaging in oral sex

The most common reasons reported by men for cunnilingus was to have fun or for pleasure (91%) followed by being in love (70%) (Table 3). Most women reported being in love (91%), followed by having fun or pleasure (60%), as their most common reasons for their first fellatio experience. Over a third of women (37%) and nearly one in ten (9%) men mentioned being married as the reason for giving their first oral sex to women. The reasons for first receiving oral sex from a sexual partner were similar. Men mentioned pleasure or fun (80%) and women (86%) mentioned being in love as the most common reasons for their first experience of receiving the oral sex from their partner.

**Table 3:** Reasons for giving or receiving first oral sex with a heterosexual partner.

Reasons for giving first oral sex to a heterosexual partner^1^	Male (n = 43)	Female (n = 35)
In love	30 (70%)	32 (91%)
Got married	4 (9%)	13 (37%)
To have fun or pleasure	39 (91%)	21 (60%)
Drunk	1 (2%)	2 (6%)
To avoid pregnancy	2 (5%)	2 (6%)
Raped or forced	2 (5%)	NR
Cajoled or pressured by partner	1 (2%)	NR
**Reasons for receiving first oral sex from a heterosexual partner** ^**1**^	**Male (n = 103)**	**Female (n = 36)**
In love	65 (63%)	31 (86%)
Got married	5 (5%)	10 (28%)
To have fun or pleasure	82 (80%)	16 (44%)
For money or seek favour	1 (1.0%)	NR
Rape or forced	2 (2%)	NR
Drunk	3 (3%)	1 (3%)
Cajoled or pressured by partner	5 (5%)	NR
To avoid pregnancy	1 (1%)	NR
During menstruation (of the female partner)	1 (1%)	NR

*1 – Participants were allowed to select multiple response; NR – Not reported by the participants*

### Factor associated with oral sex among sexually active men and women

The results of the unadjusted and adjusted models of risk factors analyses forever giving oral sex and ever receiving oral sex and ever having had oral sex (a combination of ever given and received oral sex) in men and women is shown Tables 4 and 5.

**Table 4:** Factors associated with previous report of giving or receiving oral sex among sexually active men and women from the general population in two communities in Ibadan, Nigeria (N = 626).

Variable	n/N(%)	EVER GIVEN ORAL SEX		EVER RECEIVED ORAL SEX	
		p-value^1^ Crude OR (95%CI)	p-value^2^ Adjusted OR (95%CI)^3 8^	p-value^1^ Crude OR (95%CI)	p-value^2^ Adjusted OR (95%CI)^ 8^
***(Level 1)*** ^***1***^					
***Sex***		p = 0.380	p = 0.494	p < 0.001	**p < 0.001**
Female	310/626 (50%)	1	1	1	**1**
Male	316/626 (50%)	1.24 (0.77-1.99)	1.20 (0.71-2.02)	3.68 (2.42-5.60)	**4.01 (2.52-6.38)**
***SOCIO-DEMOGRPAHIC*** ***(Level 2)***		**p-value** ^**1**^ **Crude OR (95%CI)**	**p-value** ^**2**^ **Adjusted-OR (95%CI)** ^**3 8**^	**p-value** ^**1**^ **Crude OR (95%CI)**	**p-value** ^**2**^ **Adjusted OR (95%CI)** ^** 8**^
**Age group, years**		p = 0.002	**p = 0.027**	p = 0.008	p = 0.363
18-24 years	259/626 (41%)	1	**1**	1	1
25 and above	367/626 (59%)	2.24 (1.31-3.83)	**1.89 (1.06-3.37)**	1.71 (1.14-2.55)	1.25 (0.77-2.02)
**Study site** ^**4**^		p = 0.083	p = 0.203	p = 0.021	**p = 0.028**
Ibadan North	320/626 (51%)	1	1	1	**1**
Akinyele	306/626 (49%)	0.65 (0.40-1.06)	0.72 (0.43-1.20)	0.64 (0.44-0.94)	**0.62 (0.40-0.95)**
**Ethnicity**		p = 0.487	p = 0.633	p = 0.196	p = 0.403
Yoruba	485/626 (77%)	1	1	1	1
Others	141/626 (23%)	1.22 (0.7-2.1)	1.64 (0.63-2.16)	1.34 (0.87-2.06)	0.62 (0.40-0.95)
**Religion**		p = 0.034	p = 0.177	p = 0.055	p = 0.7248
Others	275/626 (44%)	1	1	1	1
Islam	351/626 (56%)	0.60 (0.37-0.96)	0.70 (0.42-1.17)	0.69 (0.47-1.01)	1.11 (0.61-2.03)
**Highest education level**		p = 0.047	p = 0.247	P < 0.001	**p < 0.001**
None and primary level	80/626 (13%)	1	1	1	**1**
Any secondary level	401/626 (64%)	0.92 (0.43-1.98)	0.91 (0.41-2.03)	1.33 (0.68-2.57)	**0.88 (0.43-1.82)**
Any tertiary level	145/626 (23%)	1.81 (0.80-4.06)	1.46 (0.63-3.40)	3.07 (1.52-6.20)	**2.22 (1.03-4.79)**
**Occupation**		p = 0.209	p = 0.837	p = 0.011	**p = 0.053**
No current paid job^5^	111/626 (18%)	1	1	1	**1**
Paid worker	515/626 (82%)	1.54 (0.76-3.09)	1.09 (0.50-2.38)	2.03 (1.14-3.63)	**1.89 (0.97-3.66)**
**Income per month** ^**6**^		p = 0.017	p = 0.283	p < 0.001	p = 0.329
Up to 20,000N (56USD)	421/626 (67%)	1	1	1	1
More than 20,000N (>56USD)	205/626 (33%)	1.81 (1.12-2.93)	1.35 (0.78-2.32)	2.35 (1.60-3.46)	1.26 (0.79-2.01)
**Current marital status**		p = 0.739	p = 0.746	p = 0.375	p = 0.942
Living with partner	318/626 (51%)	1	1	1	1
Not living with partner^**7**^	308/626 (49%)	0.92 (0.57-1.48)	1.10 (0.62-1.93)	1.19 (0.81-1.73)	0.98 (0.60-1.61)
**Own Tv**		p = 0.722	p = 0.687	p = 0.124	p = 0.314
No	228/626 (36%)	1	1	1	1
Yes	398/626 (64%)	1.09 (0.67-1.80)	0.88 (0.48-1.62)	1.37 (0.91-2.05)	0.77 (0.45-1.29)
**Own radio**		p = 0.128	p = 0.357	p = 0.017	p = 0.959
No	299/626 (48%)	1	1	1	1
Yes	327/626 (52%)	1.45 (0.89-2.35)	1.31 (0.74-2.31)	1.59 (1.08-2.34)	1.01 (0.63-1.62)
***PROXIMATE FACTORS (Level 3)***		**p-value** ^**1**^ **Crude OR (95%CI)**	**p-value** ^**2**^ **Adjusted OR (95%CI)** ^**3 8**^	**p-value** ^**1**^ **Crude OR (95%CI)**	**p-value** ^**2**^ **Adjusted OR (95%CI)** ^**13**^
**Ever had mutual masturbation** ^**9**^		p < 0.001	**p < 0.001**	p < 0.001	**p < 0.001**
No	146/626 (23%)	1	**1**	1	**1**
Yes	480/626 (77%)	27.97 (3.85-202.89)	**23.71 (3.24-173.61)**	8.94 (3.86-20.74)	**8.10 (3.37-19.44)**
**Ever had genital circumcision** ^**10**^		p = 0.553	p = 0.804	p < 0.001	p = 0.081
No	128/626 (20%)	1	1	1	1
Yes		1.20 (0.65-2.22)	1.10 (0.53-2.28)	3.31 (1.77-6.20)	1.90 (0.91-4.00)
**Alcohol use**		p < 0.001	**p = 0.015**	p < 0.001	**p = 0.002**
No	334/626 (53%)	1	**1**	1	**1**
Yes	292/626 (47%)	2.41 (1.47-3.95)	**2.15 (1.15-4.02)**	3.46 (2.31-5.18)	**2.25 (1.33-3.81)**
**Smokes tobacco or cigarette**		p = 0.048	p = 0.311	p < 0.001	p = 0.199
No	531/626 (85%)	1	1	1	1
Yes	95/626 (15%)	1.84 (1.03-3.27)	1.43 (0.72-2.84)	3.00 (1.89-4.77)	1.44 (0.83-2.51)
**Ever had HIV test** ^**11**^		p = 0.108	p = 0.323	p = 0.208	p = 0.086
No	358/626 (57%)	1	1	1	1
Yes	268/626 (43%)	1.48 (0.92-2.38)	1.31 (0.77-2.22)	1.28 (0.87-1.86)	1.50 (0.94-2.39)
**Ever heard of HPV** ^**12**^		p = 0.067	p = 0.124)	p = 0.317	p = 0.625
No	580/626 (93%)	1	1	1	1
Yes	46/626 (7%)	2.09 (0.99-4.41)	1.96 (0.86-4.48)	1.42 (0.73-2.78)	1.22 (0.55-2.68)

***1****- p-values were obtained from Wald tests;* ***2*** *– p values were obtained from Likelihood Ratio tests;* ***3****- Level 1 factors were adjusted for level 2 and 3,;* ***4****- LGA – Local government area;* ***5****– Student, apprentice and no job;* ***6****–N –357 Naira- currency of Nigeria – 1 USD;* ***7****– Living alone;* ***8****- Level 2 factors were adjusted for sex, core variables from Level 1 and other variables in Level 2 that were significant at p < 0.10 in the unadjusted model,* ***9****- The mutual masturbation question was ‘have you or your partner ever touched each other’s genital area by hand? (Yes or No);* ***10****- Circumcision was defined as female genital mutilation or penile circumcision;* ***11****- HIV is human immune deficiency virus;* ***12****- Awareness of HPV was defined as ever heard of the infection called human papillomavirus infection (Yes or No); 13- Level 3 factors were adjusted for sex, core variables from Level 1 and level 2 factors and other variables in Level 3 that were significant at p < 0.10 in the unadjusted model*

**Table 5:** Factors associated with previous report of giving or receiving oral sex among sexually active men and women from the general population in two communities in Ibadan, Nigeria (N = 626).

Variable	n/N(%)	ANY ORAL SEX	
		p-value^1^ Crude RR (95%CI)	p-value^2^ Adjusted RR (95%CI)^3^
***Level 1***			
**Sex**	310/626 (50%)	p < 0.001	**p < 0.001**
Female	316/626 (50%)	1	**1**
Male		2.29 (1.70-3.09)	**3.12 (2.04-4.76)**
***SOCIO-DEMOGRPAHIC*** ***(Level 2)***		**p-value** ^**1**^ **Crude OR (95%CI)**	**p-value** ^**2**^ **Adjusted-OR (95%CI)** ^**8**^
**Age group, years**		p = 0.008	p = 0.234
18-24 years	259/626 (41%)	1	1
25 and above	367/626 (59%)	1.66 (1.14-2.42)	1.31 (0.84-2.05)
**Study site** ^**4**^		p = 0.091	p = 0.151
Ibadan North	320/626 (51%)	1	1
Akinyele	306/626 (49%)	0.73 (0.51-1.05)	0.75 (0.50-1.11)
**Ethnicity**		p = 0.389	p = 0.554
Yoruba	485/626 (77%)	1	1
Others	141/626 (23%)	1.20 (0.79-1.83)	1.16 (0.71-1.91)
**Religion**		p = 0.074	p = 0.816
Others	275/626 (44%)	1	1
Islam	351/626 (56%)	0.72 (0.50-1.03)	0.94 (0.53-1.64)
**Highest education level**		p = 0.002	**p = 0.014**
None and primary level	80/626 (13%)	1	**1**
Any secondary level	401/626 (64%)	1.29 (0.70-2.37)	**0.93 (0.48-1.79)**
Any tertiary level	145/626 (23%)	2.50 (1.30-4.81)	**1.82 (0.90-3.69)**
**Occupation**		p = 0.020	p = 0.105
No current paid job^5^	111/626 (18%)	1	1
Paid worker	515/626 (82%)	1.83 (1.07-3.10)	1.63 (0.89-2.97)
**Income per month** ^**6**^		p < 0.001	p = 0.349
Up to 20,000N(56USD)	421/626 (67%)	1	1
More than 20,000N (>56USD)	205/626 (33%)	2.12 (1.47-3.08)	1.23 (0.80-1.91)
**Current marital status**		p = 0.433	p = 0.934
Living with partner	318/626 (51%)	1	1
Not living with partner^**7**^	308/626 (49%)	1.15 (0.81-1.65)	1.02 (0.64-1.63)
**Own Tv**		p = 0.230	p = 0.198
No	228/626 (36%)	1	1
Yes	398/626 (64%)	1.26 (0.86-1.84)	0.73 (0.45-1.18)
**Own radio**		p = 0.014	p = 0.760
No	299/626 (48%)	1	1
Yes	327/626 (52%)	1.58 (1.10-2.28)	1.07 (0.69-1.66)
***PROXIMATE FACTORS (Level 3)***		**p-value** ^**1**^ **Crude OR (95%CI)**	**p-value** ^**2**^ **Adjusted OR (95%CI)** ^**13**^
**Ever had mutual masturbation** ^**9**^		p < 0.001	**p < 0.001**
No	147/626 (23%)	1	**1**
Yes	479/626 (77%)	9.29 (4.125-20.33)	**7.53 (3.51-16.14)**
**Ever had genital circumcision** ^**10**^		p = 0.001	p = 0.451
No	128/626 (20%)	1	1
Yes	498/626 (80%)	2.27 (1.34-3.83)	1.27 (0.68-2.40)
**Alcohol use**		p < 0.001	**p = 0.007**
No	334/626 (53%)	1	**1**
Yes	292/626 (47%)	2.84(1.96-4.14)	**1.93 (1.19-3.14)**
**Smokes tobacco or cigarette**		p < 0.001	p = 0.236
No	531/626 (85%)	1	1
Yes	95/626 (15%)	2.63 (1.67-4.14)	1.39 (0.81-2.37)
**Ever had HIV test** ^**11**^		p = 0.405	p = 0.203
No	358/626 (57%)	1	1
Yes	268/626 (43%)	1.17 (0.81-1.67)	1.33 (0.86-2.05)
**Ever heard of HPV** ^**12**^		p = 0.439	p = 0.709
No	580/626 (93%)	1	1
Yes	46/626 (7%)	1.30 (0.68-2.50)	1.15 (0.55-2.43)

***1****- p-values were obtained from Wald tests;* ***2*** *– p values were obtained from Likelihood Ratio tests;* ***3****- Level 1 factors were adjusted for level 2 and 3,;* ***4****- LGA – Local government area;* ***5****– Student, apprentice and no job;* ***6****–N –357 Naira- currency of Nigeria – 1 USD;* ***7****– Living alone;* ***8****- Level 2 factors were adjusted for sex, core variables from Level 1 and other variables in Level 2 that were significant at p < 0.10 in the unadjusted model,* ***9****- The mutual masturbation question was ‘have you or your partner ever touched each other’s genital area by hand? (Yes or No);* ***10****- Circumcision was defined as female genital mutilation or penile circumcision;* ***11****- HIV is human immune deficiency virus;* ***12****- Awareness of HPV was defined as ever heard of the infection called human papillomavirus infection (Yes or No); 9-mutual masturbation, genital circumcision, alcohol use and smoking; 13- Level 3 factors were adjusted for sex, core variables from Level 1 and level 2 factors and other variables in Level 3 that were significant at p < 0.10 in the unadjusted model*

In the adjusted model, participants aged 25 years and older had 1.89 times (95% CI, 1.06–3.37) odds of reporting ever giving oral sex compared with those aged 18–24 years (Table 4). History of ever having performed mutual masturbation (AOR = 23.71, 95%CI, 3.24–17.36) and alcohol use (AOR = 2.15, 95%CI, 1.15–4.02) were associated with higher odds of ever giving oral sex compared with those who did not report these behaviours.

Furthermore, men relative to women in the general population had 4.01 times (95%CI, 2.52–6.38) odds of reporting ever receiving oral sex. Living in peri-urban setting (Akinyele LGA) was associated with lower odds (AOR = 0.62, 95%CI, 0.40–0.95) than those living in urban setting (Ibadan North LGA). Participants with any tertiary level education had a higher odds (AOR = 2.22, 95%CI, 1.03–4.79) of ever receiving oral sex than those with up to any secondary educational level. There was a weak relationship (AOR = 1.89, 95%CI, 0.97–3.66) between participants with paid job and report of ever receiving oral sex relative to those with no current paid job. History of ever having masturbation (AOR = 8.10, 95%CI, 3.37–19.44) and alcohol use (AOR = 2.25, 95%CI, 1.33–3.81) were associated with higher odds of ever receiving oral sex. There was also a weak relationship between reporting of ever having HIV test (AOR = 1.50, 95%CI, 0.94–2.39) and history of receiving oral sex.

Concerning risk factors associated with history of ever having oral sex in the adjusted model (Table 5), men had 3.12 times (95% CI, 2.04–4.76) odds of ever reporting any type of oral sex compared to women. There was a weak positive relationship between ever reporting oral sex and having any tertiary education (AOR = 1.82, 95%CI, 0.90–3.69) relative to secondary education and below. History of ever reporting mutual masturbation was associated with higher odds (AOR = 7.53, 95%CI, 3.51–16.14) of ever reporting any form of oral sex. There was 1.93 times (95%CI, 1.19–3.14) odds of ever having any oral sex in people that had ever drank alcohol than those with no such history.

## Discussion

This study provides scientific evidence that sexually active Nigerian men and women in heterosexual relationships commonly engage in oral sexual behaviours, although at a lower reported prevalence than surveys on sexual behaviours in the UK and USA [15,20]. Generally, three out of ten men compared to one in ten women reported ever engaging in any form of oral sex with a heterosexual partner, and most were performed without using any barrier methods. There were significantly higher odds of men reporting ever having or receiving oral sex than women. However, there was no sex difference in proportions of men and women that ever gave oral sex to their heterosexual partners. Men had their first oral sex experience at younger age and had higher proportions of number of lifetime partners relative to women.

The higher prevalence of any oral sex, history of ever receiving or giving oral sex in men than women in this study are similar to most studies in high-income countries and some studies in South Africa [9,17,20,29]. Despite the similarity in framing of questions to elicit response on oral sexual practices and the observed differences in men and women in our findings with similar surveys in UK and US, the prevalence of oral sexual practices (given or received or any oral sex) in men and women were more than two folds higher in the NATSAL and NHANSE surveys than the prevalence reported in this study [15,20,30]. The higher proportion of ever receiving oral sex and engaging in any form of oral sex in men than women may be due to some of these explanations. Men generally tend to openly discuss and sometimes exaggerate their sexual activities including oral sex than women [31,32]. The same trend was reported even in high-income countries that have more liberal disposition to open discussion of sexual activities and with limited or no sex discrimination [33]. In many countries in SSA including Nigeria, men are culturally permissive to flaunt their sexual practices to portray their dominance and prowess within heterosexual relationships whereas women/girls do not enjoy such traditional rights and are shy to express themselves [33,34]. Rather, most cultural beliefs and practices in Nigeria expect women not to openly discuss their sexual behaviours and exploits including oral sexual practices [33].

In this study, the reported younger age for any pattern of oral sex initiation (ever had, and ever given or received) and higher number of lifetime oral sexual initiation in men compared with women are consistent with previous studies on oral sex and penile-vaginal sexual behaviours [35]. Generally, reports of earlier oral sexual initiation in men relative to women are associated with history of other sexual behaviours and sexual risk behaviours including engaging in unprotected sexual behaviours and multiple sexual partnership. The higher sexual risk behaviours including oral sex in men relative to women had also been associated with increasing use of alcohol, illicit drugs, and other psychoactive substances by them [36,37].

Regarding factors associated with different patterns of oral sexual behaviours, men tend to engage in receiving oral sex more than women but there is no difference in the odds of ever giving oral sex between men and women in this study. In 2009–10 NHANSE survey conducted among adults aged 20–69 years in USA, the result showed that men had 1.03 (95% CI 1.01–1.05) higher risk of ever performing oral sex than women [15]. In this same study men also had 1.84 (95% CI 1.54–2.20) higher risk of having five lifetime oral sex partners and 1.10 (95% CI 0.99–1.22) higher risk of history of performing oral sex during their first oral sex compared to women [15].

The most common reasons in this study for engaging in oral sex was being in a romantic relationship or married among men and women. It is plausible that people who engaged in oral sexual act perform it as a reflection of trust in their relationships and could possibly explain the reason why majority of them did not use barrier method/condom during the act. Previous studies including a qualitative study in Nigeria showed that people engaged in oral sex as a mark of love and to sustain their romantic relationship [33,38,39]. In addition, oral sex was also believed to be less risky than penile-vaginal and anal sexual practices.

Although we did not specifically investigate the preference of men and women on the pattern of oral sex, some studies showed that men preferred receiving than giving oral sex to women due to their fear of contracting infections including risk of mouth and throat cancers, better sexual satisfaction, and social cultural taboo against giving oral sex to women [33,40].

Some studies have associated the increased oral sexual practices in men than women to a higher risk of oral sexually transmitted infections including oral HPV infections and associated HPV-associated cancers [2,8,41]. In some African culture and religious beliefs, there are taboos that men who give oral sex are at a risk of losing their spiritual power and societal respect [34,42,43]. This cultural or religious beliefs will require further studies including qualitative design to explore the reason and interpretations of why men engage more in receiving than giving oral sex with their heterosexual partners.

We observed subtle differences in other factors associated with the pattern of oral sexual behaviours in addition to the observed sex differences between participants in this study. For example, participants with tertiary education, positive history of performing mutual masturbation, and alcohol use had higher odds of having a history of ever reporting any oral sex or ever receiving oral sex. The higher odds of oral sex in men and women with tertiary education could be due to their relatively better access to modern or “westernised” lifestyle including different sexual behaviours such as oral sex compared to those no western education [44]. In addition, there was an inverse relationship between living in peri-urban settings and history of ever receiving oral sex. The history of ever giving oral sex was associated with being 25 years and above, history of mutual masturbation and alcohol use. History of alcohol intake, use of illicit drug and ever engaging in risky sexual behaviours have been shown to be risk factors for engaging in oral sex in both heterosexual men and women [45,46]. The protective effect of living in rural/peri-urban area on oral sexual practice might be due to the general perception among some Nigerians in rural settings that oral sex is “alien” to their culture [33]. This belief might have accounted for the reduced odds of participants living in such settings. Unlike other similar studies that reported an increased risk of oral sex in younger population especially among the adolescents, however, we observed that older young adults aged 25 years and above are more likely to have had history of ever giving oral sex to their partner. It is plausible that the observed difference could be due to the variation in the age range used in these studies to categorise young people.

Unlike most studies in SSA that reported oral sexual behaviours within heterosexual relationships without using a clear definition of the sexual act, this study used a validated tool similar to the questionnaire used for NATSAL and NHANSE surveys. We translated our tool into local languages for better comprehension and to avoid any ambiguity. Therefore, we were able to distinguish reports of ever giving oral sex from history of ever receiving oral sex within heterosexual relationship among our participants. These categorizations are vital to the understanding of potential health risks associated with oral sex in men and women. We used sex matched interviewers to conduct our face-to-face interviews with our participants to minimise the risk of social desirability bias that might be associated with answering sensitive questions [47–49]. Despite these strengths, there are some potential limitations. Oral sex is not openly admitted like penile-vaginal sex especially among adult women and those that perceived oral sex as a taboo or socially unacceptable sexual practice due to cultural or religious leanings [33]. It is plausible that some of our participants with such perception or belief may have under reported their personal experience about oral sex. Men tend to over report their sexual practices to reflect dominance within heterosexual relationships whereas women often shy away from discussing their sexual experiences to avoid backlash or societal disrespect on them. Our study did not report on the pattern of oral sex among men and women involved in the same sexual relationships. Evidence suggests that oral sex may be more common in men and women involved in same sex relationship than those in heterosexual relationship [50].

## Conclusion

In conclusion, this study shows that oral sex is a common sexual behaviour among sexually active Nigerian men and women and is largely performed without using any barrier method. In heterosexual relationships, men tend to engage and report more on oral sex compared to women. The most common reason for engaging in oral sex was being in romantic relationship or married in men and women. We recommend that future studies should use mixed methods design to investigate deeper on the reasons or motivations and barriers for engaging in oral sex including those in same sex relationships. It equally important that the role of socio-cultural and religious beliefs on oral sexual practices might provide further meanings and interpretations on attitude of Nigerians towards this sexual act. The growing reports of oral sex should necessitate healthcare providers to include documenting history of this sexual act as part of the complement of sexual behaviour repertoire and its health-related consequences.

## Supporting information

S1 Checklist Inclusivity in global research. (DOCX)
